# HTLV-3/STLV-3 and HTLV-4 Viruses: Discovery, Epidemiology, Serology and Molecular Aspects

**DOI:** 10.3390/v3071074

**Published:** 2011-07-08

**Authors:** Renaud Mahieux, Antoine Gessain

**Affiliations:** 1 Equipe Oncogenèse Rétrovirale, INSERM-U758 Virologie Humaine, 69364 Lyon cedex 07, France; E-Mail: renaud.mahieux@ens-lyon.fr; 2 Ecole Normale Supérieure de Lyon, 69364 Lyon cedex 07, France; 3 IFR 128 Biosciences Lyon-Gerland, 69364 Lyon cedex 07, France; 4 Unité d'Epidémiologie et Physiopathologie des Virus Oncogènes, Département de Virologie, Institut Pasteur, 28 rue du Dr Roux, 75015 Paris, France; 5 CNRS URA 3015, Institut Pasteur, 28 rue du Dr Roux, 75015 Paris, France

**Keywords:** HTLV-3, STLV-3, HTLV-4, Tax, emerging virus, retrovirus, interspecies transmission, transformation

## Abstract

Human T cell leukemia/lymphoma virus Type 1 and 2 (HTLV-1 and HTLV-2), together with their simian counterparts (STLV-1, STLV-2), belong to the Primate T lymphotropic viruses group (PTLV). The high percentage of homologies between HTLV-1 and STLV-1 strains, led to the demonstration that most HTLV-1 subtypes arose from interspecies transmission between monkeys and humans. STLV-3 viruses belong to the third PTLV type and are equally divergent from both HTLV-1 and HTLV-2. They are endemic in several monkey species that live in West, Central and East Africa. In 2005, we, and others reported the discovery of the human homolog (HTLV-3) of STLV-3 in two asymptomatic inhabitants from South Cameroon whose sera exhibited HTLV indeterminate serologies. More recently, two other cases of HTLV-3 infection in persons living in Cameroon were reported suggesting that this virus is not extremely rare in the human population living in Central Africa. Together with STLV-3, these human viral strains belong to the PTLV-3 group. A fourth HTLV type (HTLV-4) was also discovered in the same geographical area. The overall PTLV-3 and PTLV-4 genomic organization is similar to that of HTLV-1 and HTLV-2 with the exception of their long terminal repeats (LTRs) that contain only two 21 bp repeats. As in HTLV-1, HTLV-3 Tax contains a PDZ binding motif while HTLV-4 does not. An antisense transcript was also described in HTLV-3 transfected cells. PTLV-3 molecular clones are now available and will allow scientists to study the viral cycle, the tropism and the possible pathogenicity *in vivo*. Current studies are also aimed at determining the prevalence, distribution, and modes of transmission of these viruses, as well as their possible association with human diseases. Here we will review the characteristics of these new simian and human retroviruses, whose discovery has opened new avenues of research in the retrovirology field.

## 1. Introduction

The HTLV-1, HTLV-2 and HTLV-3 human T lymphotropic viruses as well as their STLV-1, STLV-2 and STLV-3 related simian counterparts constitute the primate T-cell lymphotropic viruses (PTLV) group [1]. These different delta retroviruses share a number of epidemiological and molecular characteristics. While PTLV-1 and PTLV-2 strains have been extensively studied since the 1980s, studies on PTLV-3 are more recent and have increased in number since the discovery of HTLV-3 in 2005 [2,3]. HTLV-4, the fourth human HTLV retrovirus, was also discovered in 2005, but a simian counterpart of this virus has not been identified to date [3,4]. We will review here the characteristics of these new simian and human retroviruses, whose discovery has opened new avenues of research in the retrovirology field.

## 2. STLV-3 Discovery and Epidemiological Characteristics

**Figure 1:**
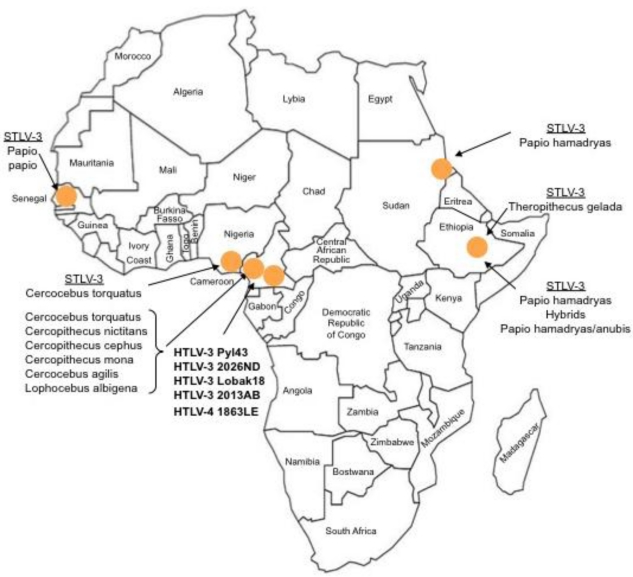
Current known geographical distribution of STLV-3, HTLV-3 and HTLV-4 viruses in the African continent. HTLV-3 and HTLV-4 are designated in bold.

The first strain of STLV-3 (strain STLV-L/PH-969) was isolated in 1994, after the long-term co-culture of human cord blood lymphocytes with the peripheral blood mononuclear cells (PBMCs), obtained from an Eritrean sacred baboon (*Papio hamadryas*) that had been kept in captivity in a research laboratory in Leuven (Belgium) [5]. The sera of two baboons tested weakly positive by immunofluorescence using HTLV-1 infected MT2 cells as a source of viral antigens and with a commercial HTLV1/2 ELISA assay. They also exhibited reactivities against the p24^gag^ and the rgp21^env^ proteins when tested with a commercial HTLV Western Blot (WB) assay. In addition, one of them reacted with the HTLV-2 gp46 specific K55 envelope peptide, resulting in a WB pattern that was indistinguishable from that of the HTLV-2 positive control [5]. The proviral sequence displayed a significant nucleotide divergence from that of the HTLV-1 and HTLV-2 prototypical sequences (40% and 38%, respectively). It was thus considered as the third lineage of the PTLV group and labeled as STLV-3. Beginning in December 2001, a series of other STLV-3 strains were then reported by several investigators, in a number of different West, Central and East African monkey species (*Cercocebus Torquatus*, *Cercopithecus nictitans*, *Papio papio*, *Theropithecus gelada*, *Cercopithecus cephus*, *Lophocebus albigena*, *Cercocebus agiles* and lastly *Cercopithecus mona)* (Figure 1). Some of these STLV-3 viruses were present in wild-caught and/or wild-born animals living in Cameroon [6–8], Nigeria [9] or Ethiopia [10]. In other cases, the animals had been kept in captivity either in France in an ethological center [11], or in US zoos [12]. Of importance, and as with the STLV-L/PH969 STLV-3 isolate, most STLV-3 serologies resembled an HTLV-2 pattern (gag^p24^ > gag^p19^ and env^gp21^ GD21, +/− env^gp46^ K55) as determined with an HTLV-1/2 commercial Western-blot assay (HTLV2-4 WB) and/or with INNOLIA kits [7–10,12]. However, in few cases, the Western-blot patterns were considered as HTLV indeterminate or “HTLV-1 like” based on the manufacturer’s criteria [12].

In comparison to STLV-1, very limited data is available on the major epidemiological determinants (*i.e.*, age, sex, modes of transmissions) of STLV-3 infection within infected non-human primates (NHP). Indeed, to date, most studies were performed in wild-caught monkeys [8,10,13,14]. The prevalence of STLV-3 infection in such studies varies greatly according to the studied NHP species, the geographical location of the animals and the methodologies used for animals sampling (living monkeys kept as pets in villages, dead animals with bush-meat samples, collection of blood or of dried blood spots). Regarding STLV-3 infection among captive monkeys, the initial paper reported that two out of 13 *Papio hamadryas*, originating from Eritrea and kept in Leuven, were infected [5]. In the study performed on *Papio papio* kept in a laboratory in France, but originating from Senegal, 4 out of the 9 baboons were found to be infected with STLV-3 [11]. Lastly, the work on the captive Gelada in US zoos indicated that 5 out of 23 animals were STLV-3 infected. In the latter group, mother-to-offspring transmission as well as horizontal transmission were suggested [12]. As is the case for the PTLV-1, a high genetic stability was also demonstrated by studying both parental and descendant strains over time [12]. STLV-3 infection has not been linked to any pathology so far. An interesting observation was made when researchers noticed that STLV-1 and STLV-3 can be found in the same primate species, as exemplified by the presence of these two viruses in *Cercopithecus nictitans*, *Cercocebus agilis*, and *Lophocebus albigena* [4,14]. A nice work performed in south Cameroon also reported cases of STLV-1/STLV-3 co-infections in the same animal [8].

**Figure 2:**
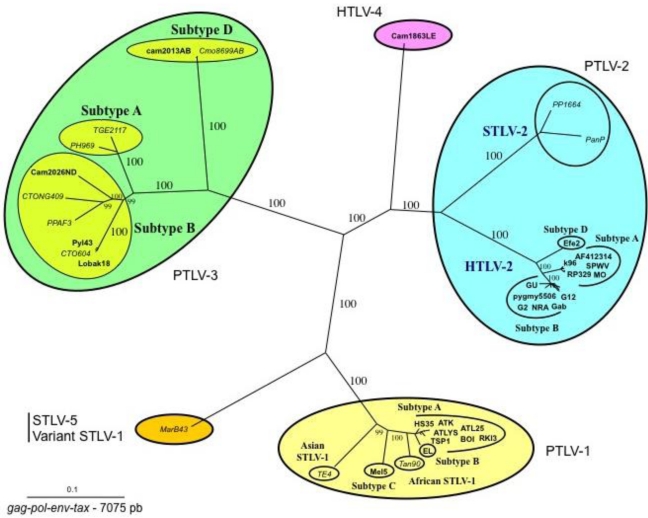
Phylogenetic tree showing the relationship between PTLV-1, PTLV-2, PTLV3, and HTLV-4. Unrooted tree generated with the neighbor-joining method, performed with the PAUP program using the 7075 bp of the concatanated *gag-pol-env-tax* genes with all the complete sequences of PTLVs available in GenBank. Branch lengths are drawn to scale. The human HTLV-3 and HTLV-4 strains are indicated in bold. Recent data based on shorter fragments of more STLV-3 strains confirmed that PTLV-3s are separated according to their geographical origin with four main distinct lineages (A, B, C, D). Mac B43 strain is a highly divergent STLV-1 originating from *Macaca arctoides*. It is considered as an STLV-5 by some authors due to its great genetic diversity compared to STLV-1/HTLV-1 strains.

Sequence comparisons of STLV-3 full-length proviruses pointed out that these strains are highly divergent from HTLV-1, HTLV-2, or STLV-2 prototype sequences (around 40% nucleotide divergence). Based on partial or complete sequence analysis, the STLV-3 lineage is now currently considered to be composed of four main subtypes (A, B, C, D) that roughly correspond to the geographical source of the strains. The subtype A comprises strains from East Africa (Ethiopia and Eritrea), the subtype B corresponds to strains from West and Central Africa, while the subtypes C and D comprise strains from Central Africa (Figure 2).

## 3. HTLV-3 Discovery, Serological and Epidemiological Characteristics

**Figure 3:**
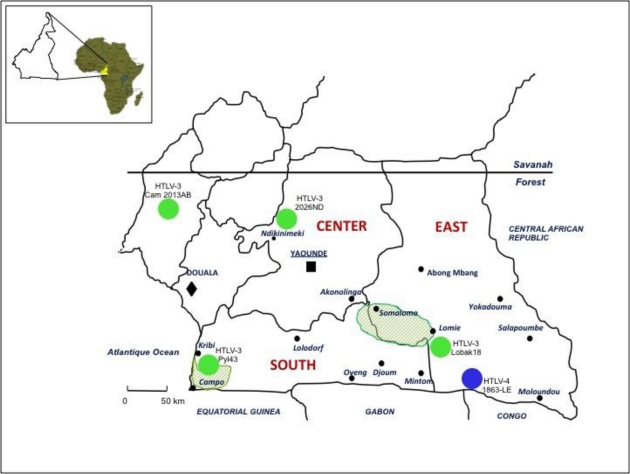
Geographical distribution of the four HTLV-3 and the unique HTLV-4 viruses reported in Cameroon. These viruses originated from individuals (either Pygmies or Bantus) living in villages or settlements of the rainforest of South Cameroon. This area is a region of high diversity for NHPs, many of them being infected by different STLV-1 and/or 3 viruses. The different human isolates are indicated with either a green circle (HTLV-3) or in blue (HTLV-4). The two shaded areas correspond to two nature reserves, the habitat of a large variety of non-human primates.

**Figure 4:**
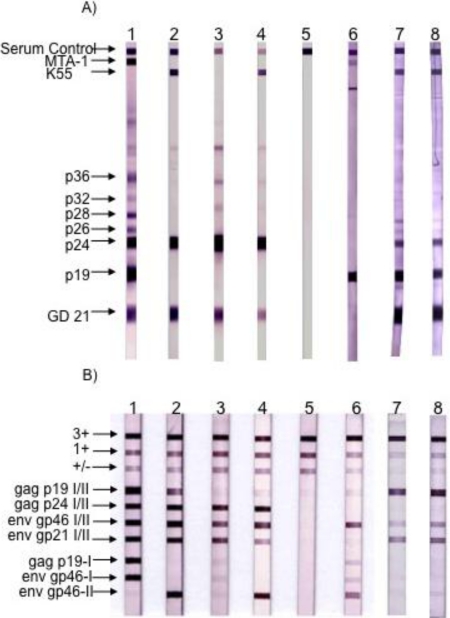
Serological pattern of HTLV-1, HTLV-2, STLV-3 and HTLV-3_Pyl43_ strain. (**A**) Western blot from MP Biomedicals (HTLV Blot 2.4 version); (**B**) Line immunoassay from Immunogenetics (INNO-LIA HTLVI/II Score). Lane 1: HTLV-1 positive control. Lane 2: HTLV-2 positive control. Lanes 3 and 4: STLV-3 positive controls. Lane 5: negative control. Lane 6: HTLV-3/Pyl43 sample. Lanes 7 and 8: Two samples of HTLV-3/Lobak18 sampled a few months apart. Infection with the other two HTLV-3 strains (2026ND and CAM2031AB) led to different Western-blot patterns when compared to HTLV-3_pyl43_ [3,17]. (Adapted from [15]).

In 2005, we and others (W. Switzer and W. Heneine's team from the Center for Diseases Control) reported the discovery of HTLV-3, a third HTLV type, in two Cameroonese asymptomatic individuals living in the rainforest area of the southern part of the country [2,3] (Table 1 and Figure 3). Their sera led to an HTLV positive serology when tested with commercial HTLV-1/2 ELISA kits, but to an HTLV indeterminate serology when tested with HTLV-1/2 commercial Western-blot assays (Figure 4). In one case (Pyl43), the WB profile displayed a clear reactivity against gag^p19^ and against the HTLV-1 env^gp46^ MTA-1 peptide [2], while in the other (2026ND), the WB showed reactivity against gag^p19^, gag^p24^, and env^GD21^ as well as a weak signal against the HTLV-1 env^gp46^ MTA-1 peptide [3]. These two viruses were not isolated in culture but their proviruses were detected using a series of PCR primers designed to amplify all known HTLVs and STLVs. More recently, the same teams reported the discovery of two additional HTLV-3 strains. The first (Lobak18) was discovered by our laboratory in another Cameroonese individual [15]. His serum also gave rise to a different WB profile, being considered as HTLV indeterminate (clear gag^p19^, gag^p24^, env^GD21^ reactivity and weak HTLV-2 gp46 env^K55^ signal). The sequence of the virus is closely related to the first HTLV-3 strain (Pyl43) [16]. Both viruses belong to the PTLV-3 subtype B. The fourth HTLV-3 strain (Cam 2013AB) was reported by the CDC team [17]. It originated from a hunter, living in Southern Cameroon, who exhibited a HTLV indeterminate WB seroreactivity. Its sequence is closely related to a divergent STLV-3 virus (Cmo 8699AB) previously found in a wild *Cercopithecus mona* from a close geographical area [4]. These two HTLV-3/STLV-3 belong to the PTLV-3 subtype D (Figure 2). The main characteristics of the four HTLV-3 strains are reported in Table 1.

An important question concerns the origin of HTLV-3 in humans. Based on the close homologies between some STLV-3 and HTLV-3 strains, and consistent with what is known for the PTLV-1 viruses, it is very tempting to suggest a zoonotic origin of HTLV-3 after interspecies transmission from STLV-3 infected NHP to humans. The diversity of HTLV-3 implies several episodes of such transmission. Exactly when these episodes occurred is currently unknown. However, the limited divergence that is observed between some STLV-3 and HTLV-3 strains (such as Pyl43, Lobak 18 and CTO 604) strongly suggests a recent non-human primate to human transmission [17]. Lastly, even if it is quite clear that the transmission *per se* occurred between hunter and NHP contact, the exact mode(s) of transmission remains speculative. Theoretically, any contact between an STLV-3-infected body fluid from a NHP and blood or muco-cutaneous tissue (with or without a lesion) could allow such zoonotic transmission. However, it is worthwhile to note that none of the four infected HTLV-3 persons ever reported a bite wound by an NHP [2,3,15,17]. This could be coincidental, or this may suggest that STLV-3 is not transmitted like the simian foamy virus (SFV), for which severe bites, whereby infected saliva and blood of the hunter most probably mix, appears as a major risk factor for transmission [18–20]. In the SFV situation, the presence of SFV in the saliva of NHPs has been well demonstrated. For STLV-3 infection, the cutting-up and butchering of NHP game may be more likely to be the route of STLV-3 transmission than bites.

## 4. HTLV-4, Another Retrovirus Found in Humans

The fourth HTLV type (HTLV-4) consists only, so far, of a unique human strain, whose provirus was also found in the PBMCs obtained from a hunter living in Cameroon [3]. The serum from the infected individual also reacted with both HTLV-1/2 ELISA and HTLV-1/2 WB commercial kits. The WB profile was that of an HTLV indeterminate serum, with the presence of a strong reactivity against the HTLV-1 gag^p19^, weak reactivities against env^rgp21^ and HTLV-2 K55 env^gp46^ peptide and no reactivity against gag^p24^ (Table 1). The complete sequence of HTLV-4 is now available. The provirus is 8791 bp long and its sequence is equidistant from HTLV-1, HTLV-2 and HTLV-3, sharing only 62–71% nucleotide identity with each of them [21]. Detailed phylogenetic analysis shows that HTLV-4 is clearly a monophyletic viral group and is considered as the oldest known PTLV-lineage. Given the previous data on PTLV-1 and PTLV-3, it is also expected that a monkey species is infected or has been infected in the past with the simian equivalent of HTLV-4. However, until now and despite the screening of a large number of monkeys from Cameroon, the search for such a virus has been unsuccessful [4,14].

## 5. HTLV-3/STLV-3 Genomic Characterization

**Figure 5:**
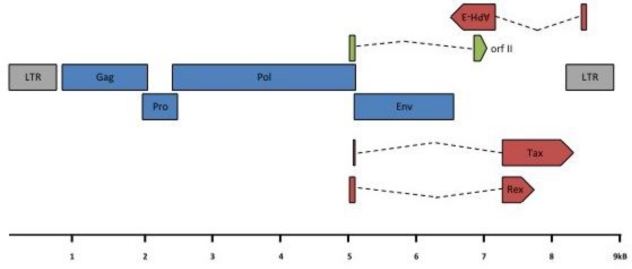
HTLV-3 genomic organization. HTLV-3 ORFs and transcription map. A scheme of the HTLV-3 genome, alternatively spliced mRNAs, and putative proteins encoded by each mRNA is shown. ORFs are indicated by boxes. *gag*, *pro*, *pol*, *env*, *tax/rex and APH-3* transcripts were amplified from 293T cells transfected with an HTLV-3 molecular clone or from STLV-3 infected cells [24,25]. Gray: LTR; blue: structural and enzymatic viral proteins; red: regulatory proteins; green: auxiliary protein. LTR: Long Terminal Repeat; (Blue color): gag: group specific antigen; pro: protease; pol: polymerase; env: envelope; (Red color): rex: regulator of expression; tax: transactivator and APH-3: antisense protein of HTLV-3; (Green color) RorfII. Note that RorfII shares its first exon with Rex.

The overall PTLV-3 genomic organization is similar to that of HTLV-1 and HTLV-2, with the presence of *gag*, *pro*, *pol*, *env, tax* and *rex* genes (Figure 5). However, LTR sequence analyses also revealed that the 5′ PTLV-3 promoter contains only two 21-bp repeats (also named Tax Responsive Element, TRE), while the HTLV-1 or HTLV-2 LTRs possess three of these sequences. The analysis of the full length HTLV-3 sequences [15–17,22] demonstrated that the HTLV-3 genome organization is similar to that of HTLV-1 and HTLV-2 and to that of central African STLV-3 strains.

HTLV-3 and STLV-3 viruses share the same genomic organization and are highly related in sequence (87 to 99% identity) [16,22,23]. Surprisingly, one of the published HTLV-3 sequences (Pyl43) contains a 366 bp deletion in its proximal pX region [16]. Importantly, this deletion should not affect Env or Tax/Rex expression. The remaining HTLV-3 published proviruses do not present this deletion. As in HTLV-1 carriers, the Tax protein is expressed *in vivo*, both in a subset of STLV-3 infected animals and in the only HTLV-3 carrier tested [23].

### 5.1. PTLV-3: Characterization of the Tax Protein

Some HTLV-3 Tax transactivator protein functions have been characterized. Even if it was shown that HTLV-1 and HTLV-3 Tax protein do not similarly bind p300, it was demonstrated that Tax1 and Tax3 proteins display a number of key similarities including the presence of a PDZ binding motif, which is absent from the Tax2 protein [22,23]. This sequence was previously shown to be critical for the ability of the Tax1 protein to transform cells *in vitro*. The results also revealed that Tax3 is functionally closely related to the transforming HTLV-1 Tax. It is indeed able to transactivate cellular promoters, to activate the NF-κB pathway, to bind the CBP transcription co-activator and can also repress the function of the p53 tumor suppressor factor. Altogether, these results suggested that HTLV-3 might be pathogenic *in vivo*. As stated above, cases of STLV-1/STLV-3 naturally co-infected animals have been described. It was therefore interesting to show that Tax3 can transactivate the heterologous HTLV-1 promoter [16,23]. Whether this could or not accelerate the occurrence of a possible disease in the co-infected animals remains to be determined.

### 5.2. PTLV-3 Auxiliary Proteins

Since its discovery, it has not been determined whether the HTLV-3 genome, as HTLV-1, encodes any auxiliary proteins that would be functionally similar to p12, p13 and p30, which are known to play important roles in the viral life cycle *in vivo*. An antisense transcript was, however, found in 293T cells transfected with an HTLV-3 molecular clone [24]. Whether this mRNA encodes a protein whose functions are similar to those of HBZ remains to be determined.

Regarding STLV-3, the presence of a doubly spliced mRNA (*RorfII*) that would encode a 64 amino acid highly hydrophobic and leucine rich protein was reported *in vivo* [24,25] (see Figure 5). The function of this protein remains unknown and should be investigated. Whether other accessory proteins are encoded from PTLV-3 genomes also remains to be experimentally determined.

### 5.3. HTLV-3/STLV-3 Receptor Complex

In contrast to HTLV-1 and HTLV-2, it was shown that the HTLV-3 surface glycoprotein (gp46 or SU) binds to both activated CD4^+^ and CD8^+^ T cells. HSPGs (heparan sulfate proteoglycans) and NRP-1 (neuropilin-1)—two molecules important for HTLV-1 entry—enhance HTLV-3 SU binding *in vitro* [26]. However, unlike HTLV-1, HTLV-3 SU can bind efficiently in the absence of both HSPGs and NRP-1. Studies of entry performed with HTLV-3 env-pseudotyped viruses together with SU binding studies revealed that the glucose transporter GLUT-1 functions at a post-binding step. Further studies revealed that HTLV-3 SU binds efficiently to naïve CD4^+^ T cells, which do not bind either HTLV-1 or HTLV-2 SU and do not express detectable levels of HSPGs, NRP-1 and GLUT-1. Altogether, these results indicated that the complex of receptor molecules used by HTLV-3 to bind to primary T lymphocytes differs from that of both HTLV-1 and HTLV-2 [26].

## 6. HTLV-4 Molecular Aspects

The molecular data on HTLV-4 is very limited. As for PTLV-3, its LTR sequence lacks the distal 21 bp repeat sequence and therefore contains only two of these elements [21]. A c-Myb binding site is also present in the viral promoter. *In silico* analyses have shown that the Tax protein from HTLV-4 lacks a PDZ binding motif, as is the case for HTLV-2. The same analyses suggested that the HTLV-4 provirus might encode 68 and 93 amino acid long auxiliary proteins. The first one, HTLV-4 ORF-IV, shares 75% similarity with the HTLV-1 p13^II^ protein, while the second HTLV-4 ORF-V displays a very weak similarity to the C-terminus of the HTLV-2 p28 protein. Finally, an ORF that would allow transcription from the 3′ LTR is also present in the HTLV-4 genome [21]. Whether these putative ORFs encode auxiliary proteins and whether this antisense transcript will functions as the HTLV-1 HBZ remains to be experimentally determined. For this reason, it seems more appropriate to name this protein APH-4 (Antisense Protein of HTLV-4) than HBZ-4. Unfortunately, neither an HTLV-4 infected cell line nor an HTLV-4 molecular clone is currently available.

## 7. Conclusions

In conclusion, we would like to reiterate four main points.

(1) The first point regards the significance of the HTLV indeterminate WB serological patterns in Central Africa [27–30] even if, as seen above, in very few cases, these reactivities are linked to the presence of a true HTLV-3 and HTLV-4 infection [2,3,31]. In each study that was performed, either in an urban or rural population, on blood donors, pregnant women or hospitalized patients, a high prevalence of HTLV WB indeterminate patterns has been reported. Such patterns are highly heterogeneous, ranging from the well-described HGIP (HTLV gag indeterminate pattern) to weak and/or isolated anti gag^p19^ or gag^p24^ reactivities [29]. Recently, another frequent indeterminate WB pattern was discovered [32]. In most cases, the significance of these WB profiles remains unknown. Indeed, in only few cases, an HTLV-1 virus could be isolated and characterized from the PBMCs of individuals whose sera displayed an HTLV indeterminate serology [33]. Several other hypotheses have been raised including the presence of defective HTLV virus, cross-reactivities with some *Plasmodium falciparum* antigens (as demonstrated in a series of cases from Cameroon) [28], or even cross-reactivity with other infectious agent antigens. Lastly, infection by another yet unknown HTLV variant or subtype might also cause such reactivities.

(2) The second point concerns the prevalence and the distribution of HTLV-3 and 4 viruses in Central African populations (Figures 1 and 3), The only data available so far were obtained from a series of a few thousand people living mostly in southern Cameroon, an area where STLV-3 infection is endemic in several monkey species. Based on these results, one could hypothesize that HTLV-3/4 viruses are also widespread (probably at a low prevalence) in other populations living in areas of Cameroon but also in other Central/West African countries such as Nigeria, Gabon, the Central African Republic, the Democratic Republic of Congo, Congo and Equatorial Guinea. Indeed, in such areas, HTLV indeterminate serologies are frequent [27–30]. However, to our knowledge, no study specifically aimed at detecting those viruses in such countries has been published. We have also to keep in mind the peculiar epidemiological situation of both HTLV-1 and HTLV-2 infection, characterized by the presence of high endemic foci, often located nearby a large region with a quite low prevalence. This has been recently exemplified in South Cameroon. Indeed, in this large area, HTLV-2 infection seems nearly absent, except in the Bakola Pygmy population [34]. Such a situation, which is very probably linked to a founder effect followed by favorable viral transmission in a specific community, may also be the case for HTLV-3 or -4 infections.

It is also important to note that there is currently no serological test available for specifically screening HTLV-3/4 viruses [31]. In fact, because of the cross-reactivities between some HTLV-1/2 and HTLV-3/4 antigens, HTLV-1/2 commercial tests are routinely used for the detection of HTLV-3 and -4. However, the sensitivity of these screening assays for detecting these viruses has not been challenged [17,31]. For this reason, specific serological screening assays are needed. The use of generic PTLV PCR primers or of HTLV-3/4 specific primers is thus necessary so far for detecting and characterizing these viruses in large series of samples with various HTLV indeterminate serologies. Obviously, such an approach is costly and time consuming, and does not allow studies on a large number of samples. Regarding the presence of HTLV-3 and -4 outside Africa, two recent studies showed the absence of HTLV-3 and HTLV-4 infection in patients from the USA, who were suffering from large granular leukemia [35,36]. Furthermore, no evidence of HTLV-3 and HTLV-4 infection was found in subjects from New York, at risk for retroviral infection [37].

(3) The third question concerns the modes of transmission of these viruses, their tropism *in vivo* and their possible associated pathology. Indeed, a case-control study based on a larger number of HTLV-3/4 positive individuals would be useful to shed light not only on the possible intra-familial transmission of these viruses but also on any possible chronic diseases and/or biological abnormalities associated with such viral infection in humans. However, one should keep in mind that, in the HTLV-1 situation, the incidence of TSP/HAM or ATL diseases is very low and follows a long latency period. If HTLV-3 infection is associated with a disease, it might therefore be necessary to follow a large cohort of infected individuals for a long period of time before one can observe any disease. Furthermore, the selection bias inherent to the enrollment of healthy persons in previous studies greatly limits the ability of identifying any acute or severe HTLV-3/4-associated diseases. A sampling conducted among hospitalized patients, including, among others, patients with neurological disorders, cancer or immunodeficiency is thus necessary, likely in Africa where the virus is known to be present. Regarding the viral tropism and the pathogenicity of PTLV-3, colonies of infected STLV-3 non-human primates are available and should allow some answers to these questions.

(4) Lastly, we would like to raise the question of the origin of HTLV-3/4 in humans. Even if it seems quite understandable that these viruses originally arose through interspecies transmission from STLV-3/4 infected monkeys to humans, it is difficult to speculate on the relative part of the direct acquisition stemming from infected monkeys *versus* the acquisition from other infected humans through sexual activity or breast-feeding. As an example, the immense majority of HTLV-1/2 infected individuals living in Central Africa, have very probably been infected through human contacts (as demonstrated by familial studies) and not from NHPs contacts. As seen above, this contrasts with foamy viruses, where most, if not all, human carriers were directly infected from foamy virus infected NHPs during hunting activities, probably mostly through severe bites [18–20]. Regarding Pyl 43 and Lobak18 HTLV-3 strains, some samples were available from spouses, children or other close familial relatives (but not parents), and all of them tested HTLV negative by serology and/or PCR studies [2,15,38]. For HTLV-4 and the two other HTLV-3 strains, no other familial samples were available to investigate the possible human-to-human transmission [3,17]. STLV-3, like STLV-1, is widespread in several non-human primate species living throughout the African continent. STLV-3 is also considered, based on current data, to have an ancient origin, greater than that for PTLV-1, but so far has only limited prevalence in humans. This is rather consistent with primary simian transfer. However, studies are ongoing to try to discover an HTLV-3 endemic population, as it is known, as seen above, for HTLV-2 in some Pygmies from Cameroon [34]. Such studies are necessary to try to obtain better insights into the origin and modes of dissemination of HTLV-3 in the African continent. Interestingly, even for some HTLV-1 subtypes and for HTLV-2 subtype B, such interrogations on their precise origin are still a matter of debate. Indeed, the simian counterpart of the HTLV-1 Cosmopolitan subtype A, which is widespread in many high endemic areas of the world (Japan, Caribbean area, South America, and others), is still not yet know. The same question remains for the origin of HTLV-2. The simian counterpart (STLV-2) of HTLV-2 is very divergent from the strains present in humans, with no evidence of recent interspecies transmission [39].

In conclusion, and as suggested recently [18,40], the increased hunting activity in a number of central African countries, including Cameroon, has increased the chances of human exposure to several infectious emerging agents, including retroviruses such as STLV, SIV and foamy viruses. This increase of hunting activities results from a combination of urban demand for bush-meat, greater access to NHP’s habitats provided in part by logging roads, better accessibility of fire arms, and finally an increase of population living in forest areas and local food needs [18,40]. Future studies will clarify whether the recently characterized HTLV-3 and HTLV-4 will emerge, or are emerging, and whether they will cause a public health hazard.

## Figures and Tables

**Table 1:** Main epidemiological and HTLV serological characteristics of the 5 individuals infected either by HTLV-3 (n = 4) or HTLV-4 (n = 1). The five infected individuals lived in the forest area of South Cameroon and were hunters of non-human primates.

**Identification**	**Age (year)**	**Sex**	**Ethnic Group**	**Screening Test**	**W.Blot HTLV 2.4**	**Inno-Lia**	**Reference**
HTLV-3 Pyl 43	62	Male	Bakola Pygmy	Weak IFA + MT2	P19, and weak MTA-1	Weak Gag p19 I/II Env gp46 I/II, Weak Gag p19 I Weak Env gp46 I Env gp46 II	[2]
HTLV-3 2026 ND	63	Male	NP	ELISA +	P19, P24, GD21, and weak MTA-1	Weak P24 I/II Env gp21	[3]
HTLV-3 Lobak 18	56	Male	Baka Pygmy	IFA + MT2	P19, P24, GD21, K55	Weak Gag P19 I/II Weak Env gp46 I/II Env gp21 I/II	[15]
HTLV-3 Cam2013AB	32	Male	Bantu	ELISA +	P24, P28, GD21		[17]
HTLV-4 1863 LE	48	Male	NP	ELISA +	P19, weak P24, P26, P28, weak GD21, weak K55.	Gag p19 I/II Gag p24 I/II Env gp46 I/II Env 21 I/II Env gp46 I	[3]
